# Evaluation of preoperative liver function test efficacy in patients with symptomatic cholelithiasis 

**Published:** 2020

**Authors:** Majid Rezaei Tavirani, Mohammad Amin Abbasi, Masoud Bagaee, Adnan Tizmaghz, Morteza Khavanin-Zadeh

**Affiliations:** 1 *Firoozabadi clinical research development unit (FCRDU), Iran University of Medical Sciences, Tehran, Iran*; 2 *Firoozgar hospital, Iran University of Medical Sciences, Tehran, Iran*; 3 *Hasheminejad Kidney Canter, Iran University of Medical Sciences, Tehran, Iran*

**Keywords:** Liver function tests, Laparoscopic cholecystectomy, Cholelithiasis

## Abstract

**Aim:**

The present study aimed at investigating the necessity of preoperative liver function tests (LFTs) in patients with uncomplicated gallstone disease before laparoscopic cholecystectomy.

**Background:**

Significant relationship between common bile duct (CBD) stones and acute cholecystitis is reported. There are contradictory reports about the effect of CBD stones on liver function tests in patients (LFTs).

**Methods:**

In the current study, patients with symptomatic cholelithiasis who referred to hospitals during January 2015 and May 2016 were enrolled. Routine tests and ultrasonography were performed on all patients before surgery. Data were presented as means ± SD and qualitative variables as frequency (percentage) were considered. Statistical analyzes were performed with SPSS software.

**Results:**

A consecutive series of 270 patients (58 males and 212 females) who referred for laparoscopic cholecystectomy were enrolled in this retrospective study. Pre- operative LFTs were normal in 249 patients (85%) and abnormal LFT was detected in 41 patients (15 %).

**Conclusion:**

This study showed that 15 % of patients with cholelithiasis without dilated CBD had impaired LFTs. Routine LFTs in preoperative evaluation of symptomatic cholelithiasis usually reveals normal findings and is not helpful in uncomplicated cholelithiasis.

## Introduction

 It is reported that common bile duct (CBD) stones appear significantly in patients with the acute cholecystitis (1). There are various laboratory tests to evaluate the simultaneous presence of the CBD stones in acute cholecystitis (2). Aspartate aminotransferase (AST) and ALT alanine aminotransferase (ALT) are generally considered as a measure of hepatocellular function, which may be impaired due to the inflammatory process. These changes may predict the presence of CBD stones and may help reduce further using invasive or expensive diagnostic modalities, such as endoscopic retrograde cholangio-pancreatography (ERCP) and magnetic resonance cholangio-pancreatography (MRCP). However, there are few studies on the impact of gallstone related disorders on liver function tests and their results are also controversial and inconsistent with each other (3-5). Recent studies have revealed that most of patients with uncomplicated acute cholecystitis have LFT within normal ranges (6). 

While the LFTs are routinely performed on all patients presenting with symptomatic cholelithiasis, few studies assessed the impacts of gall bladder stone related disorders on liver functional tests and reported varied and even controversial results (7, 8). Considering the importance of diagnostic roles of LFTs in the diagnosis of symptomatic cholelithiasis (9, 10), in the present study we aimed to investigate pre-operative LFT changes in patients with symptomatic cholelithiasis. 

## Methods

Between January 2015 and May 2016, a consecutive series of 270 patients who referred for laparoscopic cholecystectomy were enrolled in this retrospective study. We investigated patients with uncomplicated disease, whereas subjects diagnosed with complicated hepatobiliary disease, such as cholecystitis, cholangitis or pancreatitis were excluded from our study. The present study was approved by the Ethical Committee of Iran University of Medical Sciences. Informed consent was obtained from all patients. Patients were evaluated in one of the three diagnostic groups of acute cholecystitis, chronic cholecystitis and biliary colic. Patient baseline and demographic characteristics (such as age, gender) were evaluated. Pre -operative measurements included CBC, urine analysis, AST, ALT, alkaline phosphatase (ALP), total and direct bilirubin, and ultrasonography (the diameter of the CBD, gallbladder wall thickening, and the presence of gallstones) were done in all patients. Abnormal laboratory values ​​ were considered based on total bilirubin > 2 mg / dl, AST > 36 U / dl, ALT > 40 U / dl, and alkaline phosphatase > 150 U / dl. CBD diameters more than 7 millimeters were considered as an elevated diameter according to the ultrasonography findings. 

Statistical Analysis: Data were presented as means ± SD and qualitative variables as frequency (percentage). Statistical analyzes were performed with SPSS software for Windows (Statistical Product and Service Solutions, version 20.0, SSPS Inc., Chicago, IL, USA). Student t test for quantitative variables and chi-squared test for quantitative variables were used. P values ≤ 0.05 were considered as statistically significant. 

## Results

A total of 270 patients, (58 males and 212 females) who were diagnosed with symptomatic cholelithiasis after undergoing laparoscopic cholecystectomy, were recruited.

The age of patients ranged from 25-87 years old. The mean±SD age of participants was 53.82 ± 13.37 years old. Patients’characteristics are shown in Table 1. Out of 270 patients, 192 patients (71.1 %) presented with features of chronic cholecystitis, 64 cases (23.7 %) presented with features of acute cholecystitis and 14 cases (5.2 %) presented with features of biliary colic. In our study, pre- operative LFTs were normal in 249 patients (85%) and abnormal LFT was detected in 41 patients (15 %).

**Table 1:** Patient baseline characteristics

	n=270 (%)
Age (y/o)	53.82 ± 13.37
Gender (M/F)	58/212 (21.4/79.6)
Obesity (BMI> 30 kg/m2)	1068 (31.8)
Subjects	
Acute cholecystitis Chronic cholecystitis Biliary colic	192 (71.1) 64 (23.7) 14 (5.2)
Blood glucose (mg/dl)	120.89 ±27.18
AST (IU/L)	23.23 ± 10.88
ALT (IU/L)	23.06 ± 9.97
ALP (IU/L)	104.87± 28.41
Bilirubin (mg/dl)	0.97 ± 0.25

AST: Aspartate Aminotransferase; ALT: Alanine Aminotransferase; ALP: Alkaline Phosphatase

**Table 2:** Comparison of liver functional tests between groups

	Acute cholecystitis	Chronic cholecystitis	Biliary colic
AST (IU/L)	21.46 ± 8.23	26.32 ± 11.25	24.76 ± 9.34
ALT (IU/L)	23.82 ± 7.37	24.06 ± 8.97	28.36 ± 9.01
ALP (IU/L)	97.65± 24.41	102.87± 28.12	111.34± 32.35

The mean AST level was 23.23 ± 10.88 U/L that was abnormal in 12 cases (4.4 %). The mean of ALT level was 23.06 ± 9.97 U/L that was abnormal in 10 cases (3.7 %). The mean alkaline phosphatase level was 104.87± 28.41 U/L, in 18 cases (6.7 %) which were abnormal. The mean total bilirubin was 0.97 ± 0.25 mg/dL that was abnormal in 10 cases (3.7 %). As shown in table 2, LFTs were measured in three studied groups. 

Results indicate that among 41 patients with impaired LFTs, 22 patients (53.6 %) presented with features of chronic cholecystitis, 15 patients (36.5 %) presented with features of acute cholecystitis, and 4 patients (9.7 %) presented with features of biliary colic (table 3). After a 3*-*month follow*-*up, only 3 individuals out of 41 patients with abnormal LFT had an atypical biliary pathologies symptom like occasional right upper quadrant pain (symptoms recurrence).

**Table 3:** Comparison of normal or elevated liver functional tests between groups

	Acute cholecystitis	Chronic cholecystitis	Biliary colic	total
Normal LFT’s (n)	177	42	10	229
Abnormal LFT’s (n)	15	22	4	41
Number of patients (n)	192	64	14	270

## Discussion

Results revealed that 85 percent of pre-operative LFTs were normal in patients with uncomplicated cholelithiasis. The findings arein line with previous studies. Today, the necessity of many of these tests is questionable and some studies have shown that more than 90% of tests done are not indicated before surgery (11). Thapa et al. evaluated Gamma glutamyl transferase and alkaline phosphatase levels in acute cholecystitis. The results indicated that increased serum levels of gamma -GT and alkaline phosphatase can be seen in both acute cholecystitis and common bile duct stones, but increase more than 2.5 fold in alkaline phosphatase which is a predictor of CBD stones (12). Videhult et al. analyzed the diagnostic value of LFT in pancreatitis, acute cholecystitis, CBD stones and showed that the levels of bilirubin, alkaline phosphatase are the most reliable predictive factor for CBD stones, but false positive and false negative results were common, especially in patients with a history of acute cholecystitis and pancreatitis which indicating that other mechanisms are involved which are responsible for increased levels of liver enzymes in these patients (13).

In the present study, the mean age of patients was 53.82 years old, which was similar to the study of Schirmer et al, who reported 50% of patients were in the age range of 40-60 years (10). Preoperative evaluation of LFT is used to detect liver dysfunction or blockage of bile and timely property management. In the presence of preoperative LFT abnormalities, such as patients who have liver disease, laparoscopic surgery may not be a good choice for patients with liver problems and may aggravate them (14). 

In our study, about 15 % of the patients had preoperative abnormal LFT. Of this number, 18 cases had abnormal alkaline phosphatase, 12 cases had abnormal AST, 10 cases had abnormal ALT, 10 cases had abnormal total bilirubin and 7 cases had abnormal direct bilirubin. 16 patients had more than one abnormal parameter, which is often associated with abnormal ALT and AST or abnormal total bilirubin and direct bilirubin. In a study carried out by Habib and colleagues, 87.1 % of patients had normal Liver Function Test (LFT). Among these 16 patients (12.9 %) with abnormal LFT, 43.7 % of patients had acute cholecystitis, 43.7 % had chronic cholecystitis, and 12.5 % had biliary colic. In our study, of 41 patients with impaired LFT tests, 22 patients (53.6 %) had chronic cholecystitis, 15 patients (36.5 %) had acute cholecystitis, and 4 patients (9.7 %) had biliary colic (4).

According to our results routine LFTs in preoperative evaluation of symptomatic cholelithiasis usually revealed normal findings and were not helpful in uncomplicated cholelithiasis. This was similar to a previous study which indicated that abnormal LFT is the minimum predictor for cholelithiasis with a sensitivity of 22% (15). However, some studies have reported conflicting results, for example, Thapa and his colleagues in 2010 showed an increase in gamma-GT and alkaline phosphatase levels seen in both acute cholecystitis and CBD stones , but an increase of more than 2.5 fold in alkaline phosphatase is a predictor of CBD stones (12).

Previous studies in the literature have shown that using an optimal model, combining predictors such as age, jaundice, elevated ALKP, ALT, CBD dilated bile ducts or stones in the ultrasound enhanced diagnostic sensitivity and appropriate selection of patients for ERCP and cholecystectomy could be done (14, 16). In a study by Changchien and colleagues, the need for the use of ERCP in patients with symptomatic gallstones, who were candidates for laparoscopic cholecystectomy was recognized. In 115 patients diagnosed by the help of an ultrasound, ERCP and LFT were performed. In patients with normal ultrasound and normal LFT, in 97.6% ERCP were normal. ERCP in 87% of cases with an abnormal ultrasound finding also showed duct pathology and abnormality. In 17.4% of patients with normal ultrasound duct pathology at ERCP was observed. They concluded from their study that ERCP in patients with normal LFT and normal ultrasound is not necessary (17). In fact, so far no studies have investigated the relationship between liver function tests with the results of treatment and this study was actually the first study in this field. The results of the present study shows that impaired liver function tests are not good predictors of long-term evaluation of patients with cholecystectomy.

In conclusion, this study showed preoperative routine LFT measurement in patients with symptomatic cholelithiasis may not help to differentiate uncomplicated disease features. It is recommended diagnosis be made with clinical and ultrasonographicfindings in uncomplicated cases and LFT assessment focus on complicated CBD stones such as biliary pancreatitis and cholangitis.
